# Temperament and emotional overeating: the mediating role of caregiver response to children’s negative emotions

**DOI:** 10.3389/fpsyg.2024.1369252

**Published:** 2024-04-05

**Authors:** Sehyun Ju, Samantha Iwinski, Kelly K. Bost

**Affiliations:** Department of Human Development and Family Studies, University of Illinois Urbana-Champaign, Urbana, IL, United States

**Keywords:** emotional overeating, emotion regulation, caregiving, self-regulation, temperament, eating behavior, caregiver responses, negative emotions

## Abstract

**Objective:**

This study aimed to investigate the mediating effects of caregiver responses to a child’s negative emotions on the associations between infant temperament and emotional overeating in preschool children.

**Method:**

A sample of 358 children and their caregivers enrolled in the STRONG Kids 2 (SK2) birth cohort study (*N* = 468) provided data for this analysis. Caregivers completed questionnaires assessing child temperament at 3 months, caregiver response to negative emotions at 18 months, and child emotional overeating at 36 months. Structural Equation Modeling was conducted using the lavaan package in RStudio to test hypothesized models examining whether the relations between early temperament and subsequent emotional eating were mediated by caregiver responses to a child’s emotions.

**Results:**

Findings revealed that infant temperamental orienting/regulation predicted the later development of emotional overeating through supportive caregiver responses to a child’s negative emotions. Lower levels of orienting/regulation were associated with greater emotional overeating, explained by less supportive caregiver responses to the child’s emotions. Moreover, infant surgency had a positive direct influence on emotional overeating at 36 months. Both supportive and non-supportive caregiver responses to a child’s negative emotions had significant direct influences on emotional overeating.

**Conclusion:**

The results highlight the importance of caregiver response to a child’s negative emotions as a mediator between infant temperament and emotional overeating in preschool children. Intervention strategies can be implemented to support caregivers in adopting supportive responses to their child’s negative emotions to promote healthy eating behaviors from early childhood. Future studies are needed to explore these pathways of influences throughout child development.

## 1 Introduction

Emotional overeating (EOE) involves excessive food consumption to regulate negative emotions and has been observed from early childhood throughout the lifespan (Messerli-Bürgy et al., 2018; Stone et al., 2022). This eating behavior often incorporates the consumption of energy-dense foods that are high in fat, calories, and sugar, which increases the risk of obesity (Steinsbekk et al., 2016; Messerli-Bürgy et al., 2018; Favieri et al., 2021). As pediatric obesity is one of the major global health concerns, there has been growing interest in identifying early precursors of obesogenic eating behaviors, including EOE (Haycraft et al., 2011; Topham et al., 2011; Ju et al., 2022a; Stone et al., 2022). Research suggests that EOE is particularly susceptible to the influence of relational and environmental factors beyond genetics (Blissett et al., 2010; Steinsbekk et al., 2016; Ju et al., 2022a; Stone et al., 2022), as well as to child characteristics involving reactivity and regulation (Rothbart and Derryberry, 1981). In EOE, the emotions prompting regulation through overeating are typically negatively valenced, encompassing a range of sensations, such as sadness, anger, fear, and disappointment. In this study, we use the term “negative emotions” to refer collectively to these emotions, highlighting the child’s experiences and corresponding responses from caregivers. Early childhood is a pivotal period for the development of self-regulation and the establishment of eating patterns, providing an opportunity to identify associations among early precursors of EOE. However, mechanisms contributing to the development of EOE during this early developmental period remain unclear. The current study investigates associations between infant temperament and subsequent EOE and whether these associations are mediated by caregiver responses to their children’s negative emotions.

## 2 Literature review

### 2.1 Emotion and eating

Food consumption is primarily driven by one’s homeostatic motivations, marked by food approach and withdrawal behaviors that align with physiological hunger and satiation signals (Russell and Russell, 2021). However, if eating is learned to address psychological needs, the food approach behaviors may emerge regardless of physiological feelings of hunger and satiety cues (Russell and Russell, 2021). Physiological signals are often diverted by hedonic motivations, which involve activating the reward system that triggers eating for pleasure and downregulating negative emotions (Russell and Russell, 2021). This is hypothesized to be a potential mechanism through which children may acquire the pattern of food approach behavior in response to negative emotions.

Accordingly, past studies have shown that EOE is a learned response to approach food with underlying motivations to regulate emotions (Herle et al., 2018). Empirical studies have identified individual, relational, and environmental factors that are closely linked to a child’s regulation of eating under distress, with caregiver feeding practices being one of the most salient contributing factors (Blissett et al., 2010; Steinsbekk et al., 2018; Ju et al., 2022a). However, the exact mechanism of how this eating behavior develops is unknown, which demands further investigation of the influences of early precursors of EOE in preschool children. It is imperative to investigate the associations between early temperamental characteristics and caregiver responses to a child’s negative emotions as factors contributing to the development of EOE.

### 2.2 Child temperament and emotional overeating

Temperament refers to biologically based individual differences that set the foundation for underlying reactivity and regulation in the domains of affect, attention, and behavior (Rothbart and Derryberry, 1981; Rothbart, 2007). According to the psychobiological model of temperament, there are three broad dimensions of temperament: negative affectivity, orienting/regulation, and surgency/extraversion (Rothbart, 2007). Recently, there has been growing interest in investigating how temperamental reactivity and regulation may be transferable to domain-specific regulation of food intake (Saltzman et al., 2018; Russell and Russell, 2021; Ju et al., 2022b).

Negative affectivity refers to individual differences in response to external stimuli with negative emotions marked by fear, frustration, sadness, and discomfort (Rothbart and Derryberry, 1981; Rothbart, 2007). Empirical findings suggest consistent associations between higher negative reactivity to external stimuli and EOE (Messerli-Bürgy et al., 2018; Steinsbekk et al., 2018; Bjørklund et al., 2019; Ju et al., 2022a). Bjørklund et al. found that children with higher reactivity at age 6 years were more likely to engage in EOE at age 10 years only when they were low in soothability (Bjørklund et al., 2019). The greater negative emotional reactivity may indicate a greater underlying need for regulation, which triggers food approach behaviors to relieve negative emotions (Steinsbekk et al., 2018).

Temperamental orienting/regulation refers to a child’s ability to engage, maintain, and disengage attention from external stimuli, and to manage emotional responses to the stimuli (Rothbart and Derryberry, 1981; Rothbart, 2007). Orienting/regulation during infancy is known to be foundational for the later emergence of effortful control, indicating voluntary modulation of underlying reactivity (Gartstein et al., 2009). In line with the notion that emotional eating is influenced by how emotion is regulated rather than the emotion itself (Evers et al., 2010), early regulatory capacities have been shown to be inversely associated with dysregulated eating behaviors in children (Czaja et al., 2009; Steinsbekk et al., 2020; Ju et al., 2022a). For instance, children who scored lower in the ability to self-regulate emotions were more likely to engage in EOE and approach food in response to external cues (Czaja et al., 2009). However, the influence of infant temperamental orienting/regulation on subsequent engagement in EOE in early childhood has not been fully understood.

Temperamental surgency/extraversion is marked by positive anticipation and approachability to external stimuli (Rothbart and Derryberry, 1981; Rothbart, 2007). The existing literature suggests that children with high surgency may demonstrate similar approachability to food stimuli (Leung et al., 2014; Steinsbekk et al., 2020). Although there is insufficient evidence to suggest whether higher surgency predicts EOE, it has been linked to children’s food approach behaviors, including eating in the absence of hunger and external eating (Leung et al., 2014; Steinsbekk et al., 2020). However, it is still unclear how this early temperamental characteristic may contribute to the development of EOE.

### 2.3 Caregiving and child emotional overeating

#### 2.3.1 Caregiver feeding practices

Early feeding practices have been shown to impact children’s regulation of food intake (Farrow et al., 2015; Ramirez-Silva et al., 2021; Tauriello et al., 2023). Parental use of food to regulate emotions predicted greater consumption of sweets under distress in children aged 2–5 years (Blissett et al., 2010). Moreover, longitudinal research shows that feeding practices at age 6 years that use food as a reward predict EOE in children at age 8 years (Steinsbekk et al., 2016). As such, particular attention has been brought to parent–child interactions surrounding food that direct a child’s use of food for regulatory purposes (Blissett et al., 2010; Topham et al., 2011; Herle et al., 2018). However, there is insufficient evidence regarding how caregiving practices and strategies for emotion regulation, other than feeding practices, may serve as a pathway through which early temperament may translate into EOE in children.

#### 2.3.2 Caregiving practices

Previous research has examined general caregiving practices, capturing the aspects of caregiving that are influential in supporting a child’s regulation of food intake (Topham et al., 2011; Messerli-Bürgy et al., 2018). Caregiving qualities, including the caregiver’s warmth and emotional responsiveness, have been closely linked to a child’s eating behaviors, such that greater warmth and responsivity of parents/caregivers predict more regulated approaches to food in children (Topham et al., 2011). For instance, a study showed that low parental emotional responsiveness during mealtimes predicts a higher level of EOE in children when there is greater household chaos (Saltzman et al., 2019). Therefore, it is important to gain a deeper understanding of how caregivers’ emotion-related practices can influence children’s tendency to consume food to regulate negative emotions.

Early socialization of emotions encompasses a range of strategies caregivers use to address children’s negative emotions, which further cultivates self-regulation in children (Eisenberg and Fabes, 1994; Eisenberg et al., 1999). Supportive caregiver responses to a child’s emotions are known to have long-term benefits for emotion regulation, such as fostering the child’s expression of emotions and assisting them in understanding and coping with situations that elicit emotions (Eisenberg and Fabes, 1994; Zimmer-Gembeck et al., 2022). This involves caregivers validating and employing problem-solving strategies through awareness and acceptance of children’s emotional experiences (Shadur and Husson, 2019). Conversely, caregiver responses that are non-supportive of children’s emotional experiences tend to dismiss or minimize children’s emotions or punish a child’s emotional expressions, which may escalate distress and restrict emotion understanding and flexible self-regulation strategies (Eisenberg and Fabes, 1994; Zimmer-Gembeck et al., 2022). While some studies have explored emotion and eating regulation in relation to caregiver socialization of emotion, a dearth of research has examined the implications of caregivers’ responses to their child’s negative emotions on EOE in young preschool children.

### 2.4 Child temperament and caregiving practices as influences on emotional eating

Bidirectional and transactional associations between child characteristics and caregiving practices have been well documented (Sameroff, 2009). Emotion-related caregiving behaviors are susceptible to change based on caregivers’ perceptions of their children’s temperamental characteristics. A child’s ability to self-regulate emotions is influenced by early co-regulatory strategies implemented by caregivers, which, in turn, are influenced by the child’s unique needs based on biological underpinnings (Eisenberg and Fabes, 1994).

As one strategy caregivers may employ to regulate their children’s distress, feeding practices are subject to be influenced by an individual child’s characteristics (Bergmeier et al., 2014). For instance, findings suggest that parents of children with reactive temperaments and high food responsiveness are more likely to use food to help their child cope with distress (Harris et al., 2022). In addition, higher negative affectivity at 4 years was shown to predict greater emotional feeding and child EOE (Steinsbekk et al., 2018). However, less is known about the implications of infant temperament for domain-specific regulation of eating and whether caregiver behaviors surrounding children’s negative emotions affect these associations.

### 2.5 Present study

**Figure 1:**
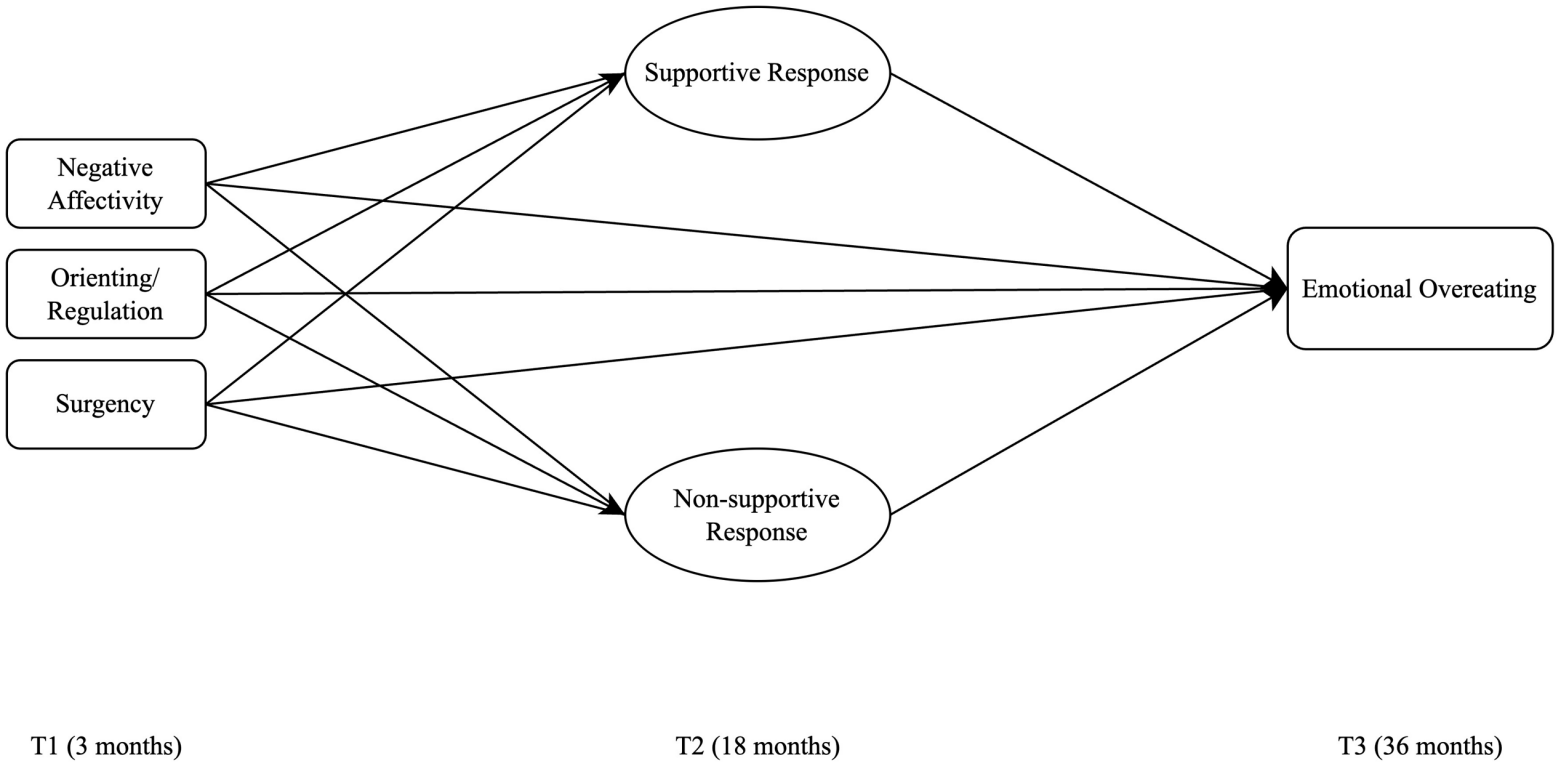
Conceptual model of the associations between early temperament and subsequent emotional eating mediated by caregiver responses to a child’s negative emotions. Statistical controls (i.e., child gender, race/ethnicity, parent education, and household income) were included in the analysis but not depicted for model simplicity.

In the present study, we aim to examine the associations between child temperament, caregiver response to their child’s negative emotions, and subsequent child EOE (Figure 1). We hypothesize that child temperament at 3 months of age has indirect effects on a child’s EOE at 36 months, mediated through caregiver responses to children’s negative emotions. Specifically, we predict that children with higher negative affectivity and lower regulation would be at greater risk of developing EOE through caregivers’ use of either a lower level of supportive or a higher level of non-supportive responses to a child’s emotions.

Although surgency is predicted to be linked to higher EOE, we could not formulate a hypothesis regarding surgency and caregiver responses due to a dearth of evidence from previous research to determine the nature of the association to be either positive or negative. Understanding early caregiver-child interactions surrounding emotions will provide valuable insights into co-regulatory approaches promoting alternative food consumption strategies that may prevent a child’s approach to food intended to regulate negative emotions.

## 3 Method

### 3.1 Participants and procedures

Participants of this study include an analysis sample of 358 children and their caregivers participating in the STRONG Kids 2 (SK2) longitudinal birth cohort study in the United States (*N* = 468; 50.6% male). Families were recruited from May 2013 to January 2017 (see Fiese et al., 2019). The primary caregiver completed validated questionnaires on the child’s eating behaviors, temperament, the caregiver’s typical response to the child’s negative emotions, and demographic information. The current analysis incorporates waves of data collected when children were at ages 3 months (Time 1 [T1]), 18 months (Time 2 [T2]), and 36 months (Time 3 [T3]). The analysis sample includes those who continued their participation until T3, with an attrition rate of approximately 7.93% from T1 to T2 and an overall attrition rate of approximately 15.20% from T1 to T3. In this analysis, 85.8% of the participants identified as White, 8.1% as Asian, 7.5% as Black, 5.0% as Hispanic/Latino, and 1.4% as Native American. Sample demographics are presented in Supplementary Table S1. The analysis sample does not differ significantly from the larger cohort on the key variables and demographics (all *p*’s > 0.05). This study received approval from the University of Illinois Institutional Review Board (# 13448).

### 3.2 Measures

#### 3.2.1 Demographic variables

Primary caregivers completed surveys on demographic information, including child age, gender, and race/ethnicity. Information regarding household income and parent education level was also obtained through caregiver reports (Supplementary Table S1).

#### 3.2.2 Dependent variable

##### 3.2.2.1 Emotional overeating

Child EOE was assessed through caregiver reports on the Child Eating Behavior Questionnaire at T3 (CEBQ) (Wardle et al., 2001). The EOE subscale includes four items assessing the child’s overeating behaviors under negative emotions (e.g., “My child eats more when worried”; α = 0.75). The items were rated on a 5-point scale (1 = *never*, 5 = *always*). The composite score was calculated such that higher scores indicate more frequent engagement in EOE (*M* = 1.68, SD = 0.58).

#### 3.2.3 Independent variables

##### 3.2.3.1 Temperament

Child temperament was assessed at T1 using caregiver reports on the Infant Behavior Questionnaire-Revised Very Short Form (IBQR-VSF; Putnam and Rothbart, 2006). The questionnaire includes 37 items with three subscales, including (a) negative affectivity (12 items; e.g., “When tired, how often did your baby show distress?”; α = 0.72), (b) orienting/regulation (12 items; e.g., “How often during the last week did the baby play with one toy or object for 5–10 min?”; α = 0.66), and (c) surgency (13 items; e.g., “How often during the week did your baby move quickly toward new objects?”; α = 0.63). The items were evaluated on a 7-point scale ranging from 1 (*never*) to 7 (*always*). The composite scores for each subscale were calculated so that the higher scores indicate greater negative affectivity, orienting/regulation, and surgency.

##### 3.2.3.2 Caregiver response to child’s emotions

Coping with Children’s Negative Emotions Scale (CCNES; Fabes et al., 2002) was used to measure caregiver responses to their children’s negative emotions at T2. The CCNES consists of 12 hypothetical scenarios in which a child expresses emotions (e.g., sadness, anger, fear, disappointment). Caregivers are then asked how they would respond to the child’s negative emotions in each scenario using six ways of coping on a 7-point Likert scale (1 = *very unlikely*, 7 = *very likely*). The items correspond to six sub-scales describing theoretically different responses caregivers use to respond to a child’s negative emotions: Expressive Encouragement Responses (EER), Emotion-Focused Responses (EFR), Problem-Focused Responses (PFR), Minimizing Responses (MR), Punitive Responses (PR), and Distress Responses (DR). In line with the theoretical framework supported by empirical evidence, the subscales were bifurcated into two overarching caregiver responses (Shadur and Husson, 2019). Accordingly, latent variables were created to represent supportive (Problem-Focused, Emotion-Focused, Expressive Encouragement) and non-supportive (Punitive, Minimizing, Distress) responses to children’s distress.

### 3.3 Analysis plan

Structural Equation Modeling (SEM) was conducted using the lavaan package 0.6–12 in R 4.1.2. The model tested supportive and non-supportive caregiver responses to a child’s negative emotions as potential mediators of the associations between child temperament and EOE. We conducted confirmatory factor analyses of the latent variables on supportive and non-supportive caregiver responses to a child’s negative emotions at T2. The model fit indices, including CFI, TLI, RMSEA, and SRMR, were assessed to obtain and improve the model fit. Then, we conducted structural regression with bootstrapping (*n* = 5,000) to test the pathways of associations between child temperament, caregiver response to a child’s negative emotions, and EOE. The demographic variables that have been shown to explain variance in children’s eating behaviors from the previous research (i.e., child gender, race/ethnicity, parent education, and household income) were included in the model as statistical controls (Powell et al., 2017; Favieri et al., 2021).

## 4 Results

### 4.1 Preliminary analysis

Missing data analysis using Little’s MCAR test revealed that the data in the analytic sample was assumed to be missing completely at random (*χ^2^*[63] = 67.59, *p* = 0.32). The missing values were handled using Full Information Maximum Likelihood (FIML) procedures. Descriptive statistics and bivariate correlations of the key study variables are presented in Table 1.

**Table 1:** Descriptive statistics and correlations for study variables.

	Mean	*SD*	1	2	3	4	5	6	7	8	9
1. Negative affectivity (IBQR-VSF)	3.53	0.84	–								
2. Orienting/regulation (IBQR-VSF)	5.39	0.67	−0.10^*^	–							
3. Surgency/extraversion (IBQR-VSF)	3.66	0.91	0.11^*^	0.40^**^	–						
4. Expressive encouragement responses (CCNES:EER)	5.28	1.12	0.48	0.13^**^	0.06	–					
5. Emotion-focused responses (CCNES:EFR)	5.67	0.91	0.02	0.15^**^	0.09	0.49^***^	–				
6. Problem-focused responses (CCNES:PFR)	5.76	0.86	0.04	0.18^***^	0.05	0.65^***^	0.75^***^	–			
7. Minimizing responses (CCNES:MR)	2.15	0.80	0.01	0.05	0.12^*^	−0.30^***^	−0.01	−0.11^*^	–		
8. Punitive responses (CCNES:PR)	2.01	0.70	0.02	−0.04	0.08	−0.29^***^	−0.12^*^	−0.17^**^	0.75^***^	–	
9. Distress responses (CCNES:DR)	2.58	0.66	0.09	−0.16^**^	−0.08	−0.27^***^	−0.16^*^	−0.29^***^	0.31^***^	0.47^***^	–
10. Emotional overeating (CEBQ)	1.68	0.58	0.07	−0.05	0.11^*^	−0.16^**^	−0.09	−21^***^	0.20^***^	0.18^***^	0.18^***^

IBQR-VSF, Infant Behavior Questionnaire-Revised Very Short Form; CCNES, Coping with Children’s Negative Emotions Scale; CEBQ, Child Eating Behavior Questionnaire. *p < 0.05, **p < 0.01, ***p < 0.001.

### 4.2 Confirmatory factor analyses

**Figure 2:**
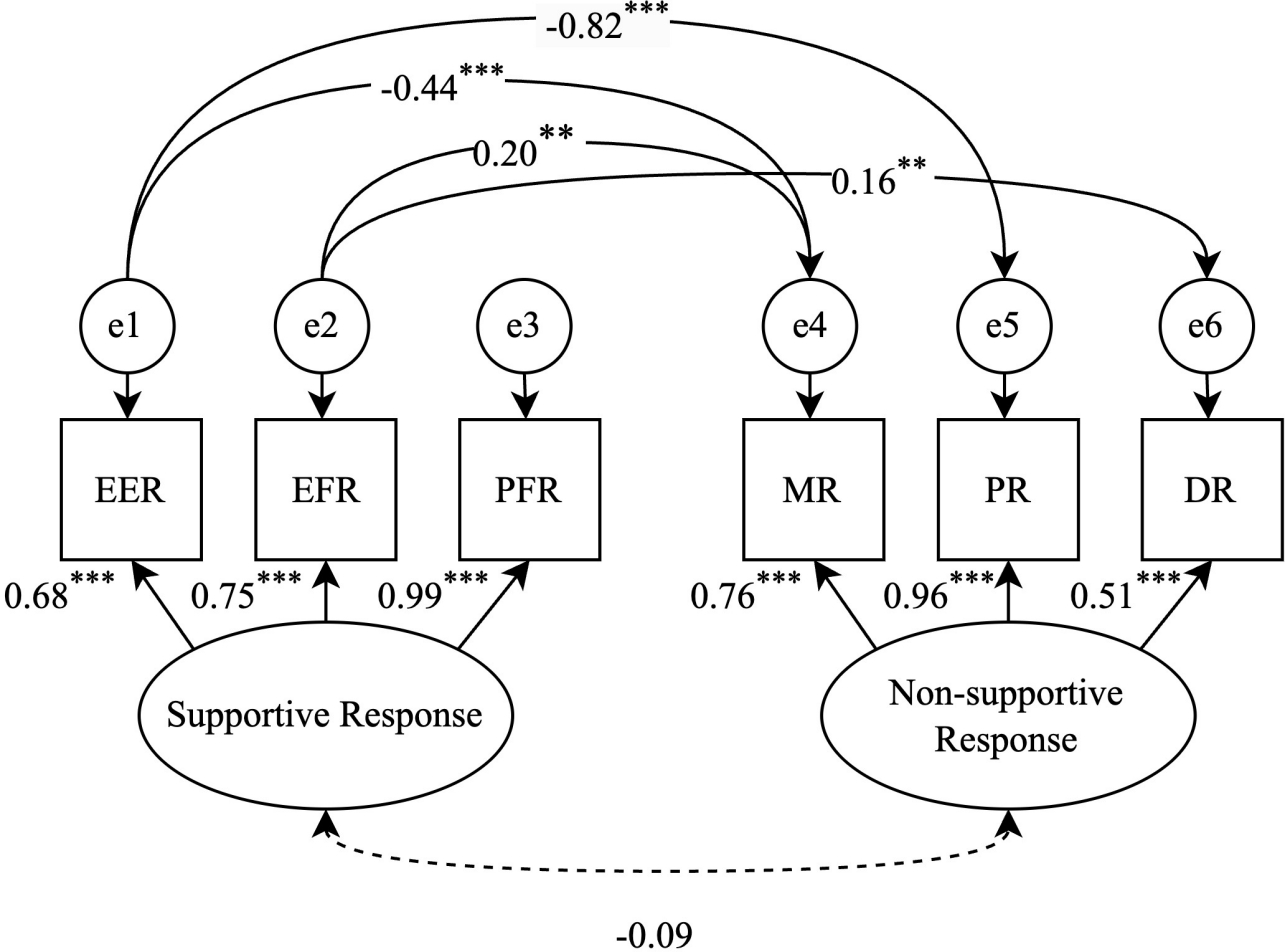
A measurement model for caregiver responses to child’s negative emotions. EER, Expressive Encouragement Responses; EFR, Emotion-Focused Responses; PFR, Problem-Focused Responses; MR, Minimizing Responses; PR, Punitive Responses; DR, Distress Responses. The initial model fit demonstrated poor fit to the data with fit indices *χ*^2^(8) = 59.72, *p* < 0.001, CFI = 0.943, TLI = 0.894, RMSEA = 0.134, SRMR = 0.075. The model was revised to include covariances between the error terms of EER, EFR, MR, PR, and DR to account for shared method variance, resulting in an improved fit: *χ*^2^(3) = 7.05, *p* = 0.070, CFI = 0.996, TLI = 0.978, RMSEA = 0.061, SRMR = 0.035. The figure presents the standardized factor loadings for the indicators of ‘Supportive Response’ and ‘Non-supportive Response. ^**^p < 0.01 and ^***^p < 0.001.

Confirmatory factor analysis (CFA) was conducted to verify the construct of caregiver response to a child’s negative emotions, encompassing two latent variables: supportive and non-supportive caregiver responses. Although individual items demonstrated robust factor loadings, the initial model exhibited a poor fit to the data (*χ*^2^[8] = 59.72, *p* < 0.001, CFI = 0.943, TLI = 0.894, RMSEA = 0.134, SRMR = 0.075). After reviewing the modification indices, residual correlations were suggested between the error terms of the following items: ‘EER’ and ‘MR,’ ‘EER’ and ‘PR,’ ‘EFR’ and ‘MR,’ and ‘EFR’ and ‘DR.’ The negative correlation of ‘EER’ with ‘MR’ and ‘PR’ aligns with theoretical expectations, as it is plausible that caregivers who use expressive encouragement may be less likely to punish or minimize a child’s negative emotions. Conversely, emotion-focused responses may be accompanied by tendencies to minimize the child’s distress with attempts to reduce the intensity of emotions as well as greater negative emotional responses in caregivers. These correlations were added to the model to account for the shared variance between the items. The modified measurement model had a good fit (*χ*^2^[3] = 7.05, *p* = 0.070, CFI = 0.996, TLI = 0.978, RMSEA = 0.061, SRMR = 0.035; Figure 2), with all indicator variables loading highly onto the respective factors (standardized factor loadings ranging from 0.51 to 0.99). The results revealed no significant association among latent variables (*r* = −0.09, *p* = 0.22).

### 4.3 Structural equation modeling

**Figure 3:**
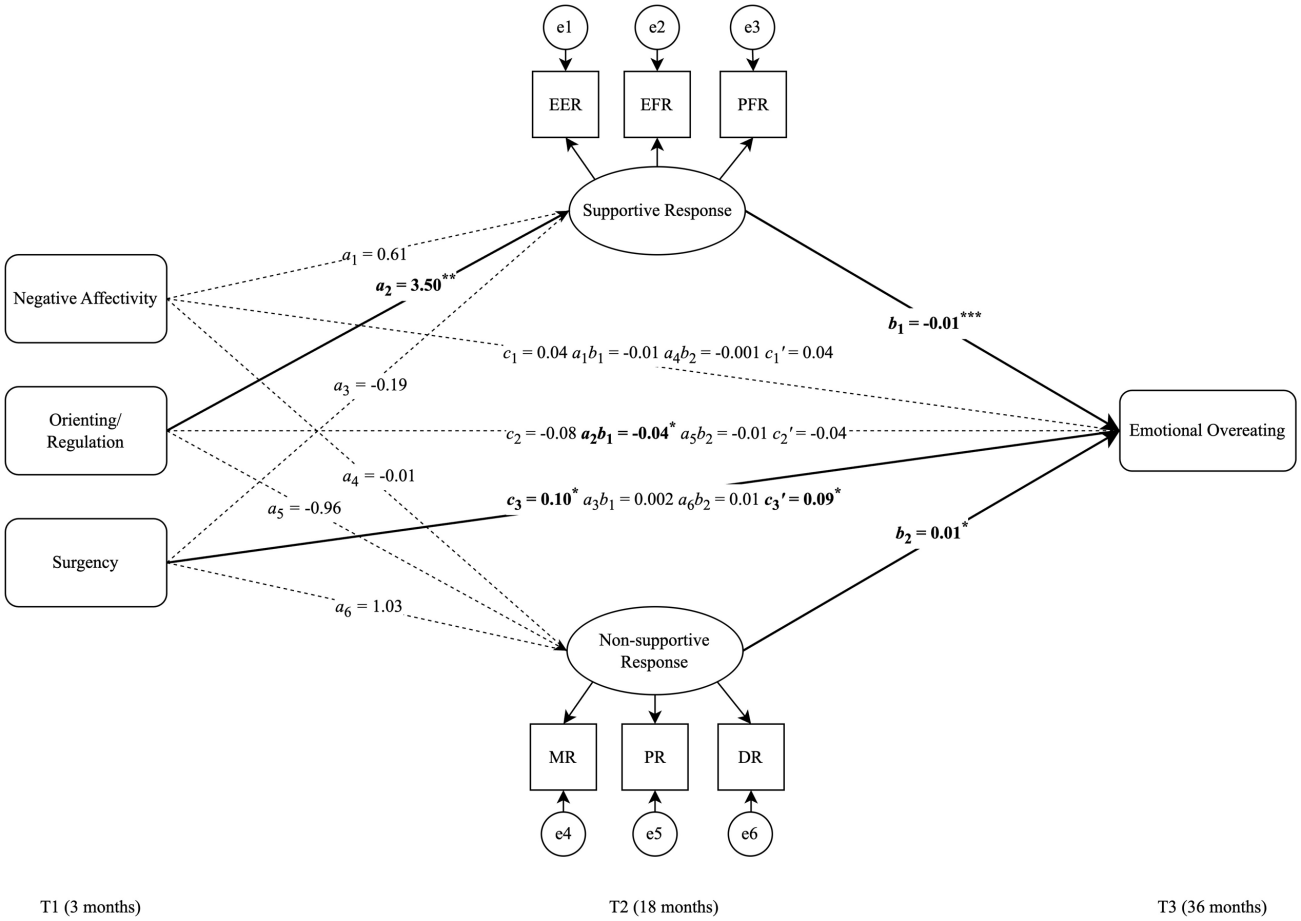
Model 1: A mediation structural model for supportive and non-supportive caregiver responses to a child’s negative emotions. EER, expressive encouragement responses; EFR, emotion-focused responses; PFR, problem-focused responses; MR, Minimizing Responses; PR, Punitive Responses; DR, Distress Responses; T1, Time 1; T2, Time 2; T3, Time 3. Model fit: *χ*2[104] = 144.84, *p* = 0.005, CFI = 0.959, TLI = 0.945, RMSEA = 0.033, SRMR = 0.043. Path coefficients *a, b, c,* and *c*’ represent unstandardized estimates (^*^p < 0.05, ^**^p < 0.01, ^***^p < 0.001), with *c* and *c*’ indicating the total and direct effects, respectively, while the *ab* coefficient indicating the indirect effects. Bold lines show statistically significant pathways, and dashed lines indicate non-significant paths. Estimates are statistically adjusted for the child’s gender and race/ethnicity, parent education, and household income. Covariances among variables were not depicted in the figure for simplicity.

The structural regression model demonstrated an excellent fit to the data (*χ^2^*[104] = 144.84, *p* = 0.005, CFI = 0.959, TLI = 0.945, RMSEA = 0.033, SRMR = 0.043), providing a robust framework to examine the direct and mediated effects (Figure 3).

#### 4.3.1 Direct effects

The SEM analysis revealed that child temperamental surgency at T1 had a significant positive effect on EOE at T3 (*c*_3_ = 0.09, SE = 0.04, *p* = 0.03), adjusting for child gender, race/ethnicity, household income, parent education, and other variables included in the model. Additionally, there were significant positive associations between orienting/regulation and supportive caregiver response to negative emotions (*a*_2_ = 3.50, SE = 1.11, *p* = 0.002). Supportive caregiver response to a child’s negative emotions was a significant predictor of EOE, such that an increase in supportive caregiver response reduced EOE in children (*b*_1_ = −0.10, SE = 0.003, *p* < 0.001). Conversely, the non-supportive caregiver response was directly related to an increase in children’s EOE (*b*_2_ *= 0*.01, SE = 0.003, *p* = 0.04). Early child temperament did not have significant direct effects on caregiver use of non-supportive responses to a child’s negative emotions.

#### 4.3.2 Mediation effects

Supportive caregiver responses to a child’s distress at T2 significantly mediated the association between T1 orienting/regulation and T3 EOE (*a*_2_*b*_1_ = −0.04, SE = 0.01, *p* = 0.02, 95% CI [−0.067, −0.010]). The pathway indicates that higher levels of orienting/regulation predict higher levels of supportive responses, which, in turn, are associated with lower EOE in children. Non-supportive caregiver responses at T2 was not a significant mediator of the associations between child temperament and EOE, but rather had a significant direct effect on EOE.

## 5 Discussion

This study examined prospective associations between child temperament, caregiver responses to their child’s negative emotions, and child EOE. In line with the literature suggesting the role of parenting practices in the development of overeating behaviors (Stone et al., 2022), we found that infant temperamental orienting/regulation predicts later development of EOE through supportive caregiver response to a child’s negative emotions. Notably, biologically based temperament dispositions in infancy had distinctive pathways of influence on later EOE. Orienting/regulation in infancy indirectly predicted EOE through its influence on caregiver responses to a child’s emotions, while surgency demonstrated a direct effect on EOE, independent of the caregiver responses. Although early child characteristics were not directly associated with caregivers’ subsequent use of non-supportive responses to the child’s negative emotions, caregiver non-supportive responses were significantly associated with EOE.

The current study suggests that less supportive and more non-supportive caregiver responses to a child’s distress have unique influences on EOE in children. These findings are consistent with the literature suggesting that the caregiver–child relationship, as one proximal influence within a child’s ecology, is influential in the development of regulatory capacities in children (Eisenberg and Fabes, 1994; Zimmer-Gembeck et al., 2022). As children gain self-regulatory abilities, caregivers play integral roles in socializing strategies for emotion and eating regulation (Steinsbekk et al., 2018). Therefore, a supportive response to child distress may be crucial in how infant temperament affects domain-specific regulation of food intake in response to negative emotions. This is especially important because EOE involves regulating both emotion and eating.

The current study highlights the importance of temperamental characteristics that may increase supportive responses from caregivers, thus reducing the risk of developing EOE in early childhood. In response to a child’s temperament, marked by low orienting/regulation, a caregiver may implement less supportive responses to a child’s emotions, which may, in turn, facilitate a child’s approach to food for regulatory purposes. These less supportive responses may be influenced by caregivers’ early challenges in utilizing supportive responses to soothe their children who exhibit low levels of orienting/regulation, having difficulty disengaging from stressors and reorienting their attention toward neutral stimuli (Rothbart and Derryberry, 1981). Furthermore, children’s development of EOE in response to caregivers’ less supportive response may be partially attributable to the lack of effective emotion regulation strategies or the implementation of maladaptive strategies that require EOE to be adopted as a means for emotion regulation (Arnow et al., 1995; Spoor et al., 2007). Tan and Holub (2015) discussed how caregivers’ use of food as a coping mechanism may not only reflect their challenges with the regulation of their own emotions but also how they might extend this strategy to manage their children’s distress. As children and adolescents lacking adequate emotion regulation strategies are at increased risk of engaging in overeating behaviors (Favieri et al., 2021), the lower level of supportive strategies from caregivers that validate children’s emotional experiences and support regulatory processes may further prompt children to utilize food for emotion regulation.

Higher infant temperamental surgency was associated with an increased risk of developing EOE in preschool age. This aligns with existing research suggesting high surgency to be associated with other behavioral attributes such as hyperactivity, impulsivity, and externalizing behaviors, which may lead to impulsivity in eating behaviors (Karreman et al., 2010). While some studies suggest that its impulsive aspect can prompt less sensitive caregiving, surgency is also marked by positive emotionality, closely linked with positive caregiving practices (Bridgett et al., 2013). Nonetheless, our findings did not reveal a link between surgency and either supportive or non-supportive caregiver responses to a child’s negative emotions, indicating that the impact of surgency on EOE may operate independently of these caregiving dimensions. Moreover, it remains unclear which aspects of infant temperamental orienting/regulation may prompt caregivers to respond to their child’s negative emotions with less supportive strategies, in contrast to surgency, which directly influences EOE. Therefore, further investigation is needed to identify aspects of a child’s temperamental reactivity and regulation (i.e., activity level, impulsivity, soothablity) that may elicit distinct caregiver responses to a child’s emotions, which may predict the later development of EOE.

Contrary to our hypotheses and the existing literature, child negative affectivity did not predict subsequent EOE directly or through the effect of caregiver responses to negative emotions (Steinsbekk et al., 2018; Tauriello et al., 2023). It is possible that the inclusion of surgency and orienting/regulation in the model might have attenuated the direct influence of negative emotionality on EOE. It may be that the specific influence of caregivers on the association between early negative affectivity and later EOE may be contingent upon the caregiver’s implementation of strategies that specifically instill the value of food as serving regulatory purposes (Steinsbekk et al., 2018; Stifter and Moding, 2018; Stone et al., 2022). Along with our results revealing the influence of temperamental orienting/regulation on EOE through the increase in supportive caregiver response to negative emotions, this may suggest that it is not the emotion itself but rather how it is regulated that has implications for the use of food as a means of self-regulation (Evers et al., 2010).

### 5.1 Strengths, limitations, and future directions

The present study provides insights into the regulatory mechanism underlying EOE by identifying mediating pathways between individual and relational early childhood factors that are associated with subsequent EOE. The current study entails an assessment of the key variables across different time points, tracing the pathways of associations during this critical period of development when children go through rapid changes in gaining self-regulatory capacities. At this early age, developing the capacity to regulate eating behaviors may be more susceptible to the influences of the intricate interplay between child temperament and caregiving practices. Recognizing this early susceptibility holds particular importance because early childhood eating behaviors have long-lasting health implications. This highlights the need for early intervention and prevention efforts accounting for child characteristics and caregiver behaviors. Potential intervention approaches could focus on modifying caregiving behaviors, enhancing caregiver understanding of their child’s temperament, and improving caregiver responses to children’s emotions to promote regulated eating behaviors in children.

Despite these strengths, the study is not without limitations. We have employed the term “negative emotions” to describe emotions such as fear, anger, and sadness, which is in line with the conventional usage in the literature. However, we acknowledge that this term may inadvertently minimize the functional and adaptive roles of these emotions in developmental processes. Therefore, the careful interpretation of the term “negative emotions” in our findings is essential, considering them as a range of emotional states with diverse implications for child development. Moreover, the majority of primary caregivers identified by the participating families were mothers, with an insufficient representation of fathers or non-parent caregivers to capture different caregiver roles in the association. This may influence the generalizability of our findings to other caregiver-child dynamics, which highlights the need for future research to include a broader range of caregiver perspectives.

In addition, the majority of our sample identified as White and were well-educated, which precludes generalization of the findings to families and individuals from different racial, ethnic, and socioeconomic backgrounds. The approaches caregivers adopt to support children’s expression of negative emotions are influenced by sociocultural factors, which may prompt parents to use suppression as a strategy to cope with children’s expressions of distress. For example, caregivers of children who are identified as Black may use suppression approaches as adaptive, supportive strategies with which children may control their emotions in preparation for systemic racism and biased teacher perceptions (Leerkes et al., 2015; Dunbar et al., 2022). Future research should examine the associations in diverse samples to understand the influence of child characteristics and caregiving practices on emotion-induced eating in children from different racial, ethnic, and cultural backgrounds.

The findings rely on caregiver reports, and the associations found may be influenced by shared method variance or a common informant bias. Findings should be replicated in future investigations using multiple methods (observational measures, laboratory-based tasks) as well as multiple informants. However, parental reports and laboratory assessments of temperament using the IBQ have been shown to be correlated (Parade and Leerkes, 2008). It is also important to note that the internal consistency of orienting/regulation and surgency subscales of the IBQR, while previously established as valid and reliable (Parade and Leerkes, 2008), was slightly lower than the commonly accepted threshold of 0.70 in the current sample. Thus, future replication studies are needed to examine the nature of these associations. Despite the extensiveness of our study using a longitudinal design, we cannot completely rule out the possibility of spurious influences of unobserved factors. Future studies should identify specific aspects of child characteristics and caregiving practices that may contribute to the development of a tendency to turn to food for affect regulation.

## 6 Conclusion

Temperament phenotypes involving reactivity and regulation have implications for children’s EOE, but some of the relations are indirect or mediated by how caregivers respond to their children’s negative emotions. Our findings suggest a caregiver’s influence on emotion regulation as one potential mechanistic pathway of association between child characteristics and later development of EOE. Results also highlight the need to examine different temperament dimensions and both supportive and non-supportive caregiver coping strategies in examining children’s self-regulation of eating. Further investigations are needed to trace the changes in these pathways of influences across development. Moreover, education and prevention aimed at helping caregivers cope with their children’s negative emotions in response to temperament characteristics should be implemented to prevent the early development of overeating behaviors in response to distress discordant with one’s physiological needs.

## Data availability statement

The raw data supporting the conclusions of this article will be made available by the authors, without undue reservation.

## Ethics statement

The studies involving humans were approved by University of Illinois Institutional Review Board (no. 13448). The studies were conducted in accordance with the local legislation and institutional requirements. Written informed consent for participation in this study was provided by the participants’ legal guardians/next of kin.

## Author contributions

SJ: Conceptualization, Data curation, Formal analysis, Investigation, Methodology, Software, Validation, Visualization, Writing – original draft, Writing – review & editing. SI: Conceptualization, Data curation, Validation, Writing – review & editing. KB: Conceptualization, Data curation, Funding acquisition, Methodology, Project administration, Resources, Supervision, Validation, Writing – review & editing.
