# Supplementary material for: Temperament and emotional overeating: the mediating role of caregiver response to children’s negative emotions
Source: Front Psychol. 2024 Apr 5;15:1369252. doi: 10.3389/fpsyg.2024.1369252 (PMC11026708; doi:10.3389/fpsyg.2024.1369252)
Supplement: Supplementary file 1 [file Table_1.DOCX]

Supplementary Material

Table S1.

*Demographic characteristics for analysis sample.*

|  | *n* | % |
| --- | --- | --- |
| Child Gender |  |  |
| Male | 181 | 50.6 |
| Female | 177 | 49.4 |
| Child race/ethnicity | |  |
| White | 307 | 85.8 |
| Asian | 29 | 8.1 |
| Black | 27 | 7.5 |
| Hispanic/Latino | 18 | 5.0 |
| Native American | 5 | 1.4 |
| Household income |  |  |
| $3000 and under | 72 | 22.7 |
| $3001–$5000 | 112 | 35.3 |
| $5001 and above | 133 | 42.0 |
| Parent education |  |  |
| Some high school | 1 | .3 |
| High school graduate | 10 | 2.8 |
| Some college or technical school | 61 | 17.2 |
| College graduate | 119 | 33.6 |
| Post-Graduate work | 163 | 46.0 |
